# The promise, problems and pitfalls of mass drug administration for malaria elimination: a qualitative study with scientists and policymakers

**DOI:** 10.1093/inthealth/ihy079

**Published:** 2018-11-03

**Authors:** Nils Kaehler, Bipin Adhikari, Phaik Yeong Cheah, Nicholas P J Day, Daniel H Paris, Marcel Tanner, Christopher Pell

**Affiliations:** 1 Swiss Tropical and Public Health Institute, Basel, Switzerland; 2 Mahidol Oxford Research Unit, Faculty of Tropical Medicine, Mahidol University, Bangkok, Thailand; 3 Centre for Tropical Medicine and Global Health, Nuffield Department of Medicine, University of Oxford, Oxford, UK; 4 Ethox Centre, Nuffield Department of Population Health, University of Oxford, Old Road Campus, Oxford, UK; 5 Amsterdam Institute for Global Health and Development (AIGHD), Amsterdam Health Technology Centre, Tower C4, Paasheuvelweg 25, BP Amsterdam, The Netherlands; 6 Centre for Social Science and Global Health, University of Amsterdam, Nieuwe Achtergracht 166, WV, Amsterdam, The Netherlands

**Keywords:** community engagement, elimination, governance, malaria control, policy

## Abstract

**Background:**

The emergence of artemisinin resistance in the Greater Mekong Subregion (GMS) has prompted urgent containment measures. One possible approach is mass drug administration (MDA). This article explores attitudes towards and perceptions of MDA for malaria elimination among policymakers and leading malariologists.

**Methods:**

Thirty-two semistructured interviews (SSI) were conducted with policymakers (n=17) and principal investigators (n=15) selected based on their involvement in malaria prevention, control and elimination in the GMS. Interviews were audio recorded and transcribed for qualitative content (thematic) analysis using NVivo (QSR International, Doncaster, Victoria, Australia).

**Results:**

Researchers and policymakers described reluctance and consequently delays to pilot MDA for malaria elimination. Most policymakers and some researchers reported concerns around the evidence base, citing a lack of data on its effectiveness and appropriate target populations. There were also worries about promoting resistance. Other issues included a previous lack of support from the World Health Organization, past MDAs, the remoteness of target populations and challenges explaining the rationale for MDA.

**Conclusions:**

The complex rationale for MDA for malaria elimination, mistaking pilot studies for implementation, past experiences with MDA, difficulties in selecting appropriate sites and the WHO’s lack of clear backing undermined the support for MDA for malaria elimination.

## Introduction

Over the last century, malaria parasites have developed resistance to every antimalarial.^1^ Quinine, one of the first antimalarials discovered, was used as the only available drug for *Plasmodium falciparum* outside of Asia for slightly less than 300 y, when finally resistance was described in 1911.^2^ Later, resistance to chloroquine developed within only 12 y from its first use as a treatment in 1945. Resistance to proguanil developed within 1 y of its initial use in 1948.^3^ Subsequently, pyrimethamine, which was introduced in 1952, could not be used as monotherapy due to cross-resistance with proguanil,^2^ and resistance to sulfadoxine–pyrimethamine (SP) developed within the same year of its introduction in 1967.^3^ Resistance to mefloquine, which subsequently replaced SP in 1977 as a first-line treatment, also developed within 5 y.^4^ During the 1990s, resistance to atovaquone developed within 1 y of its use.^5^ Since 2004, artemisinin combination therapy (ACT) has been in use as a first-line treatment in many countries. Resistance to ACT was first reported in 2008.^6^ Currently there are no alternative antimalarials to replace ACT.

The emergence of artemisinin-resistant malaria in the Greater Mekong Subregion (GMS) has added urgency to efforts to eliminate *falciparum* malaria and contain the spread of resistant strains towards Africa.^7–11^ The threat of a public health emergency if multidrug-resistant malaria reaches Africa has prompted scientists to consider approaches to accelerate elimination in addition to the basic control tools currently used in malaria prevention and control programmes.

In low to moderate transmission areas of the GMS, scientists are evaluating mass drug administration (MDA) as part of a multipronged approach to malaria elimination.^12–14^ This strategy, termed targeted malaria elimination (TME),^1^ aims to interrupt the transmission of *falciparum* malaria from foci where artemisinin resistance has been identified. In TME, all members of the target communities, whether infected or not, are offered antimalarial treatment. Recent pilot studies of TME consisted of dihydroartemisinin piperaquine (DHA-PPQ) for 3 d and a single low-dose primaquine every month for 3 months. A recent systematic review on the safety and efficacy of DHA-PPQ showed that there was no increased risk with its use.^15^ The MDA is complemented by rigorous implementation of conventional malaria control activities, including the distribution of long-lasting insecticide-treated bed nets.^1,16^ The MDA approach is widely used to control several neglected tropical diseases, including lymphatic filariasis.^17^

In the past, MDA has been used in malaria control programmes, but its wider uptake in policy has been limited by the perceived risk of drug resistance^18^ and difficulties with implementation, specifically the requirement of high coverage in target communities.^1,19,20^ The most prominent concern about frequent and large-scale MDA is the potential to increase selective pressure on the parasite and thus accelerate the emergence and spread of drug resistance. In the case of *falciparum* malaria, such reservations are compounded by the prospect of resistance to artemisinin-based therapies, which are generally the first-line treatment.^1^ Several strategies, as used in TME, are available to mitigate the emergence of resistance: 1. target patients with very low parasite densities (subclinical infections) and 2. reduce the time period between rounds of MDA to reduce the fitness advantage for novel resistant *falciparum* strains or the amplification of already resistant strains.^1^

Concurrently, to maximize the chance of interrupting local malaria transmission, models indicate that coverage in target communities must be >80%.^16,19,21^ Particularly in the isolated and mobile communities where transmission is focused throughout the GMS, this is a logistical challenge.^22–30^ Based on such perceived risks, policymakers have been hesitant to support MDA for malaria prevention and control.

Considering the urgent need for accelerated malaria elimination in the GMS, it is critical to explore the current views and perceptions towards MDA. However, little has been reported regarding the views of key stakeholders towards MDA for malaria elimination: how do policymakers and malariologists view MDA as an approach? How important are concerns about the inadvertent spread of resistance? What do they see as the main implementation challenges? What evidence would require policymakers to implement MDA as a malaria elimination strategy? Drawing on in-depth interviews, this article explores the attitudes of policymakers and leading malariologists towards MDA for malaria elimination.

## Methods

Data collection was conducted between October 2016 and April 2017 at various locations in Thailand, Myanmar, Cambodia, Laos, Vietnam and the USA.

### Respondents

Respondents were recruited based on their expertise (principal investigators of current malaria elimination studies and senior malariologists) or decision-making roles in malaria prevention, control and elimination in the GMS (policymakers in Thailand, Cambodia, Myanmar, Vietnam and Laos, and funders, such as from the World Health Organization [WHO] and the Bill and Melinda Gates Foundation) (Table 1). Potential respondents were identified through a combination of a snowball approach and bibliography and web searches. A snowball approach was used to overcome the difficulties contacting and setting appointments with respondents who have busy schedules. The appropriateness of potential respondents identified during interviews or web searches was discussed among core members of the research team.

**Table 1.** Characteristics of respondents

Policymakers	Principle investigators
17	15
8 were national and 9 were international	2 were national and 13 were international

The contact details of potential respondents were obtained from institutional websites or from other respondents. Potential respondents were subsequently contacted by e-mail. None of the potential respondents explicitly refused to participate; two potential participants did not respond to the approaches or failed repeatedly to keep arranged appointments. Neither offered a reason for this.

Semi-structured interviews (SSIs) were conducted until a point of theoretical saturation was reached, i.e. when no novel information emerged from the data.^31^ Seventeen interviews were conducted with policymakers/funders and 24 were conducted with study principal investigators/senior malariologists.

### Data collection

Whenever possible, interviews were conducted face to face at one of the TME study sites, selected international tropical medicine conferences or at the ministerial offices of the respective country. If a face-to-face meeting was not possible, Skype or telephone interviews were conducted. Two respondents were not available for an interview and responded to an e-mail questionnaire.

All interviews were conducted in English by the first author. Interview length ranged from 20 to 90 min. On average, interviews with policymakers and funders were longer than those with scientists. Interviews were audio recorded and transcribed verbatim by an independent transcriber and all transcripts were checked for quality.

During the interviews, a semistructured interview guide was used to direct questioning. Topics included malaria elimination, MDA and community engagement. Under these broad topics, a flexible and iterative approach was taken to questioning to elicit in-depth information and to ensure that relevant topics were not neglected.

### Data analysis

The interview transcripts were analysed using qualitative data analysis software (NVivo 11; QRS International, Burlington, MA, USA). A codebook adapted from previous qualitative research on TME and community engagement (in Cambodia, Laos and Myanmar) was used. Line-by-line coding of transcripts was conducted using these pre-established themes (deductive approach, e.g. malaria elimination, community engagement, MDA), followed by themes that emerged during the data analysis (inductive approach). Analysis continued by identifying and explaining prominent themes, patterns among respondents and outliers. To ensure coding reliability, all coding was initially done independently and then collectively discussed and agreed on. Emerging themes were discussed among members of the study team before further integration.

### Ethics approval

Ethical approval was obtained from the Oxford Tropical Research Ethics Committee (OxTREC) and approved on 31 January 2017 (Unique Protocol ID: OxTREC ref: 5122-16). Verbal informed consent was obtained from all respondents prior to interviews.

## Results

Respondents included eight policymakers from six countries and nine representing international health institutions. Nine of the principal investigators/senior malariologists were connected to recent TME pilot studies of MDA for malaria elimination in the GMS region and six were connected to other international research institutes in the USA, UK, Japan and Australia.

**Figure 1.**
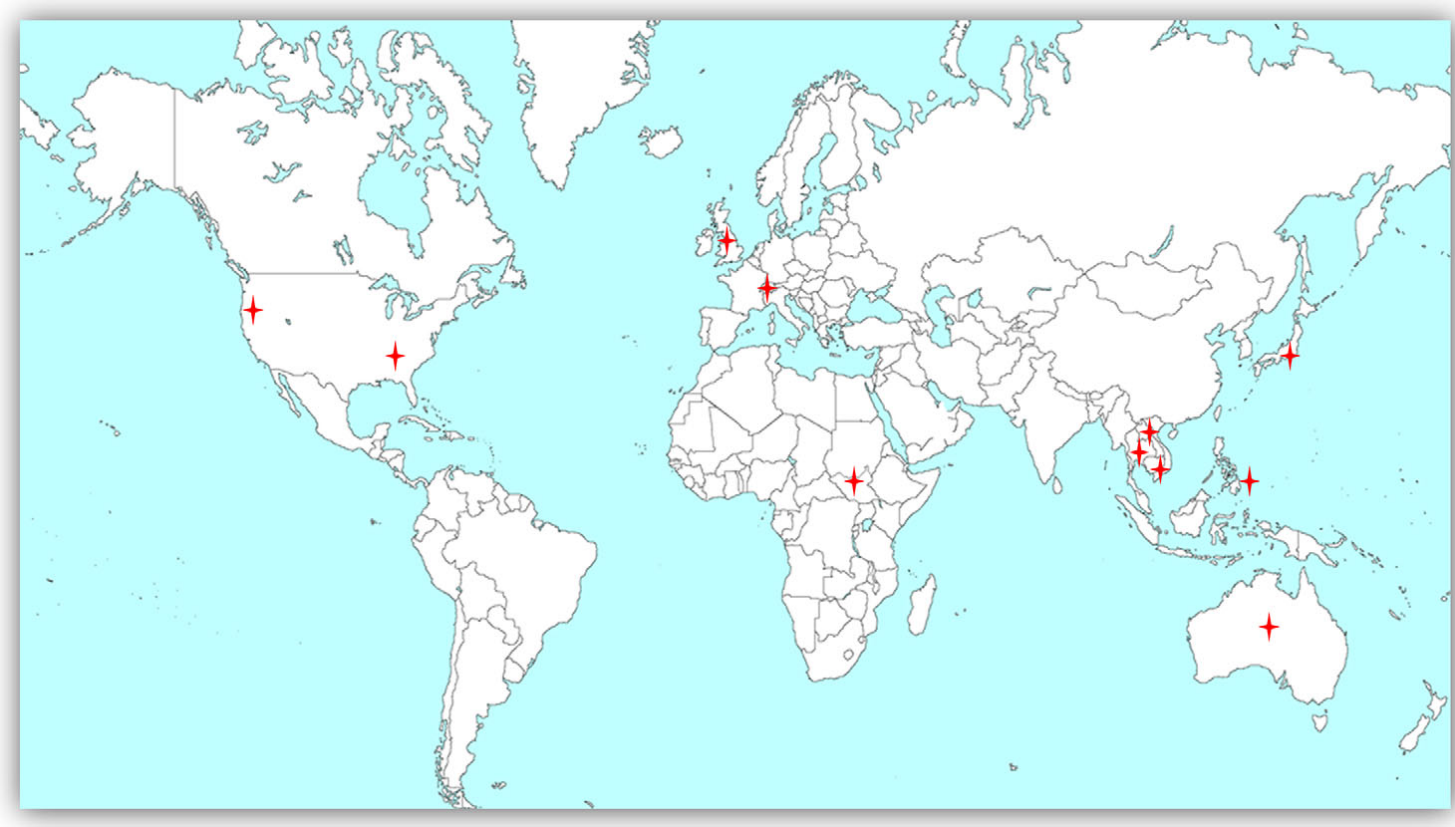
Geographic diversity and distribution of respondents in the study (red marks represent respondents’ locations).

**Figure 2.**
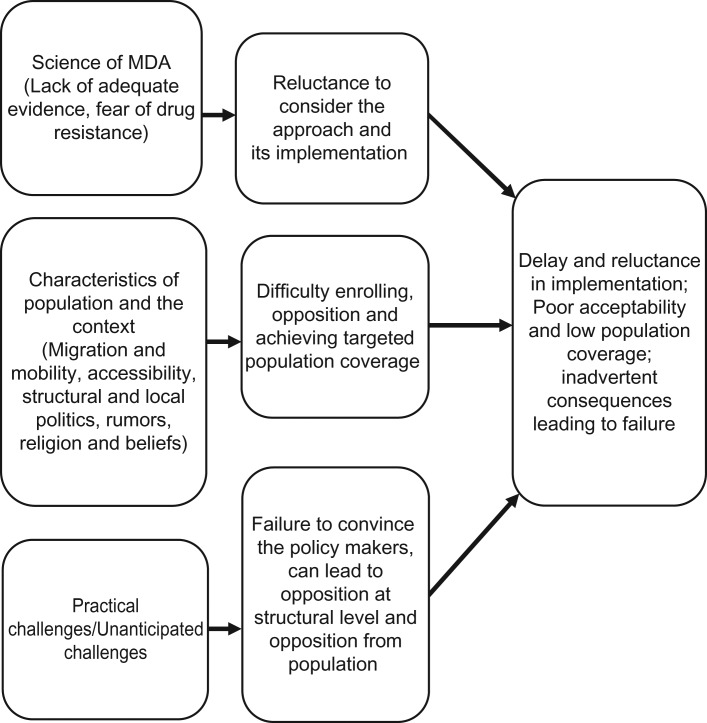
Suggested factors affecting implementation and acceptability of MDA.

Scientists and policymakers discussed their experiences and familiarity with MDA (Figure 1) and the challenges associated with its implementation, including acceptability (Figure 2). All respondents were familiar with MDA, mostly as a tool for controlling other tropical diseases, such as microfilariasis, schistosomiasis and helminthic diseases. Sixteen respondents were familiar with the TME pilot studies.

Most respondents described concerns about malaria elimination through MDA. They raised four types of issues related to 1. the science of MDA, its rationale and possible ramifications (amount of work involved, safety concerns and resistance); 2. national and local politics; 3. appropriate locations for MDA, including population mobility; and 4. how to roll out MDA.

### The science of MDA, its rationale and possible ramifications

Most policymakers and some researchers described concerns around the evidence base for MDA for malaria control and elimination. Although potentially a useful tool, they cited a lack of evidence regarding its effectiveness and appropriate circumstances, particularly in terms of malaria transmission dynamics.

Scientists highlighted the importance of choosing the right drug combination for effective MDA. With regard to possible drug combinations for MDA, most researchers reported that DHA-PPQ (as used in TME) was appropriate, because there was enough safety evidence. Some researchers suggested that, in the future, increasing resistance to current drugs will necessitate the use of three antimalarials in combination. Others considered existing combination therapy sufficient for the elimination of low-density, subclinical infections. Triple therapy may be more appropriate for treatment of clinical infections with higher parasite densities.

In the TME studies, single, low-dose primaquine to suppress gametocytaemia was used in addition to DHA-PPQ (with the exception of Cambodia, where regulatory approval to use primaquine was not given). Respondents recognized that using primaquine for radical therapy of *Plasmodium vivax* malaria (14 d course) in glucose-6-phosphate-deficient populations, however, can potentially cause severe haemolysis. Some scientists mentioned the potential use of tafenoquine, referring to the ease of administering it as a single-dose regimen. However, serious concerns about haemolysis were expressed about this drug as well, as it has a long half-life and once given cannot be ‘stopped’. In addition, the lack of safety data on tafenoquine further restricts its use and potential role in the future.

Most scientists and policymakers described a fear of MDA increasing drug resistance and affecting the drugs available for future treatment. The scientists involved with the recent studies of MDA for malaria elimination in the GMS questioned the assumption that increased drug use would lead to increased drug resistance. Specifically, they described how administering a complete treatment course is unlikely to increase the risk of resistant strains emerging or becoming amplified.

Policymakers were unclear about the rationale for MDA as a strategy to prevent the spread of resistance. According to some researchers, the potential catastrophic outcome of the spread of resistant parasites was not taken as seriously as the circumstances require.

We failed to convince them [the policymakers] that it is urgent. And so I think it’s too late now because resistance has continued to spread and it’s probably now going to follow its natural course to India, Bangladesh and then Africa.

—SSI with a malariologist (#27)

### Political challenges

Most respondents described political challenges at all levels: from the highest level of policymaking to the target communities. Researchers described primarily challenges connected to studies of MDA, whereas most policymakers referred to challenges in connection with MDA implementation within malaria control programmes. In regard to studies of MDA, political challenges were described from the initial phase of seeking ethical approval to the final steps before study initiation. Seeking government approval was considered critical but time-consuming. A factor mentioned to influence this process was the selection of key policymakers (for instance, members of the ministry of health and heads of departments of parasitology and malaria) responsible for decision making.

In general, scientist respondents linked the delays to a misunderstanding about the purpose of the TME pilot studies, with policymakers regarding them as a strategy to implement MDA rather than research studies to assess efficacy and feasibility. The lack of policy support due to apparent misunderstanding varied across the countries. At the national level, scientists reported unknown factors underpinning governments’ support or opposition to an MDA trial, particularly because sometimes decisions changed suddenly without any specific reason.

We talked about mass drug administration, and people like the head of the malaria control programme was adamantly against it, and other people were also kind of saying, ‘Over my dead body,’ basically, but that changed within two years…they didn’t give any specific reasons for it. It’s kind of a belief system.

—SSI with a malariologist (#32)

There were less ambiguous reasons underpinning some decisions regarding national-level MDA research. One country had reportedly decided to support an MDA study, yet when a former but still influential politician suddenly opposed the study, the decision was reversed. Several researchers blamed the WHO’s initial lack of support for MDA as prompting several GMS states to delay MDA trials. In Myanmar and Vietnam, where the government had already approved an MDA study, the opposing parties suddenly objected to studies, citing the lack of a WHO recommendation.

Political division in communities reportedly also impacted the success of several MDA studies. One researcher described how along the Thai–Myanmar border, strong political divisions and refusal to participate in MDA by one half of the community who were affiliated with a particular political party detrimentally affected the study outcome.

### Appropriate locations for MDA

#### Mobility and migration

Respondents were concerned about the mobility of target populations, which often cross national and international borders in search of work, health care and security. This was considered a prominent challenge to interrupting malaria transmission. Community members who are busy with agricultural work (mostly rice plantation) in the fields during rainy seasons can miss part of all components of the MDA and were viewed as presenting a specific challenge.

High-risk populations, living and working in and around forests, where malaria has been linked to occupations, such as logging, hunting and farming, were also seen as a particular challenge. One scientist pointed out that transmission often does not occur within the villages but more commonly in the forests, requiring that subjects would have to pass through such sites and get infected before they can subsequently spread further the infection within the village. In regard to the impact of mobility on MDA, the risk of new infections is therefore lower than anticipated, as not all mobile subjects have passed through an area of transmission and therefore do not import new malaria parasites to the community.

Geographically confined territories, such as islands, were considered more appropriate sites for MDA because of the ease of monitoring mobility in target communities.

SSI-P25: I said what I would prefer is to go to a road, take the end of the road, 10 villages and do it there. The only thing that you have to control is this [mobility]. There will be less migration [mobility].

To overcome challenges of mobility, one policy maker suggested massively scaling up the target areas but at the same time emphasized the consequential need for mobilizing significant resources. Several scientists were confident that the challenge posed by mobility can be overcome by a well-functioning detection and treatment system.

#### Remoteness and isolation of target communities

Scientists and policymaker agreed that selecting a site appropriate for MDA was a prominent challenge. It was generally agreed that MDA is effective in low-transmission settings, including in urban areas.

In the GMS, transmission often occurs in forested regions, which are difficult to access. This remoteness was reported to have caused difficulties with recruitment of staff willing to undertake the necessary long and difficult journeys, accept arduous working hours and live under basic living conditions for days or weeks. Also, when choosing sites for the pilot studies, remote, villages with poor accessibility had to be excluded as the blood sample collection and processing had to be completed within 24 h and could not be performed on site.

### How to roll out MDA

#### Acceptability in target communities

One respondent described how a rumour started by a single person could apparently affect population coverage in the pilot studies. In addition, negative experiences with past MDAs were described as having often caused problems. For instance, in Cambodia, several people became ill after an MDA some years ago, and this prompted a strong aversion to MDA in local communities (and among policy makers). Stories of a death linked to an MDA in Myanmar had a similar impact.

SSI with a senior policy maker (#12): …that there was an MDA project I think back in the end of the last decade in Myanmar and there was a death basically, …he was saying that at that moment people were saying let’s NOT go for MDA, ....there was such a push back because publicity was like you can kill people, so let’s not move in that direction.

Researchers raised the importance of community engagement to respond to such issues, and study staff could talk to members of target communities and address directly their concerns. Some researchers mentioned that inadequate attention to community engagement (that entailed helping the target population to understand the study rationale and outcome) can jeopardize the MDA. Others referred to a lack of staff and representatives at the community level to answer and respond to such rumours. Indeed, most researchers viewed the collaboration of community members, through active training and jointly executing the MDA, as a key element of community engagement. Amongst the manifold benefits of community collaboration, the sense of ownership felt by the community in jointly undertaking MDA was described as critical.

Some respondents also highlighted the complexity of the concept of asymptomatic malaria infection and the difficulties that the target populations had in understanding this. Hence, motivating local community members who were seemingly healthy to participate in MDA was recognized as a difficult task.

Also, the presence of staff to monitor and address adverse events during and after MDA was considered crucial to avoid potential drop-outs and rejections. Researchers added that this was relevant regardless of whether the ‘perceived side effects’ were directly related or otherwise to the drugs.

#### Seasonal timing of MDA

Seasons reportedly had a double impact on MDA. Around the rainy season (just before or after), when malaria incidence is highest, is regarded to be the best time to perform MDA in terms of clearing parasites. However, in this period, many villagers are occupied with agricultural work and may be unavailable for several weeks. Additionally, during the rainy season, access to the villages is often severely compromised due to worsening of road conditions.

SSI with a malariologist (#30): It’s very difficult in [this country]. Many—the road system has improved in my time here, but there are still—in the rainy season, quite large communities—difficult to get into and resupply because of landslides and overflowing rivers, etc.

In the rainy season, seasonal illnesses, such as acute respiratory tract infections, were sometimes misinterpreted as side effects of the MDA drug. This was compounded if supervision and consultation after MDA was insufficient.

## Discussion

This is the first article to explore policy makers' and leading malariologists’ views on research into and the use of MDA for malaria elimination. The findings highlight different opinions between policy makers and researchers. Although most respondents echoed the challenges associated with implementation of MDA, national and international policy makers were particularly sceptical about applying this strategy for malaria elimination. The findings highlight several areas for discussion: 1. the inhibitory influence of the WHO on policy makers when forming their opinions; 2. the impact of previous MDAs in influencing attitudes to MDA, which were often intertwined with local politics; and 3. challenges identifying appropriate sites for MDA.

### Evidence and the role of the WHO

Several policy makers were unclear about the rationale for MDA as a tool for malaria elimination. This was in spite of the topic having been discussed by senior researchers, governmental officials, and NGO staff during at least one international meeting every year in the GMS and at every ASTMH conference between 2013 and 2017 (Table 2). Two major systematic reviews of clinical research on MDA have been published,^19,21^ and two have addressed the role of community engagement in MDA^16,32^ (Table 3). Also, in late 2015, for the first time, the WHO published a recommendation on the use of MDA in malaria elimination based on a recent review of evidence^33^ and the advice of the Malaria Policy Advisory Committee which took place in April 2015.^34^

**Table 2.** Summary of meetings and published documents on MDA before the interviews

Year	Event	MDA-related content	Participants
2013	Meeting for operational research on malaria elimination, October 2013, Geneva, Switzerland	Meeting: discussion of the optimal MDA strategy (i.e. timing, duration, combinations of drugs, number of treatment rounds, demographically and geographically defined at-risk populations, monitoring of adverse events and population acceptability)	Senior researchers and government officials
2013	ASTMH Annual Meeting, Washington DC, USA, November 2013 (Available at: http://www.abstractsonline.com/Plan/ViewSession.aspx?sKey=2ae4d000-e718-4bce-8464-20085506c849&mKey=%7bCEAFE81A-9B33-4623-A1BB-85D31108B94B%7d)	Symposium: Implementation of Mass Drug Administration for Malaria Control and Elimination (7 presentations)	Senior researchers, government officials, NGOs; open to the public
2014	National Malaria Research Network of Cambodia Workshop, July 2014	Update on clinical studies: MORU presented its experiences with TME along the Thai–Myanmar border. TME was found to be best way to target villages effectively	Senior researchers and government officials
2014	Workshop on Targeted Mass Treatment for Malaria Elimination, Phnom Penh, November 2014	Workshop MDA/TME	Senior researchers, government officials, NGOs
2014	ASTMH Annual Meeting, New Orleans, LA, USA, November 2014 (http://www.abstractsonline.com/Plan/SSResults.aspx)	Symposium: Recent Experiences of Using Targeted Mass Treatment with Antimalarial Drugs for Parasite Clearance from the Human Reservoir in Southeast Asia and Africa (6 presentations)	Senior researchers, government officials and NGOs; open to the public
2015	ASTMH Annual Meeting, Philadelphia, PA, USA, October 2015 (http://www.abstractsonline.com/Plan/ViewSession.aspx?sKey=a6ecff33-a023-4961-8be7-a7cecae1b56c&mKey=%7bAB652FDF-0111-45C7-A5E5-0BA9D4AF5E12%7d)	Symposium: Current Lessons from Drug-Based Strategies for Malaria Elimination	Senior researchers, government officials and NGOs; open to the public
2016	Mass Drug Administration WHO Policy Update (Global Malaria Program), May 2016, Bogota, Colombia (http://www.paho.org/hq/index.php?option=com_docman&task=doc_view&gid=34878&Itemid=270&lang=pt)	Dr A. Bosman, Global Malaria Program and WHO: ‘The new WHO recommendations on MDA’	Senior researchers, government officials and NGOs
2016	ASTMH Annual Meeting, Atlanta GA, USA, November 2016 (http://www.abstractsonline.com/pp8/#!/4114/)	Sessions: Malaria: Epidemiology I—Intervention Studies and Evaluation (session 5) Malaria Elimination Strategies Using Targeted Mass Drug Administration: Lessons from the Field (session 55) Integration of Mass Drug Administration with Vector Control Approaches: An Enhanced Malaria Elimination Package (session 96)	Senior researchers, government officials and NGOs; open to the public

ASTMH: American Society of Tropical Medicine and Hygiene; MORU: Mahidol-Oxford Tropical Medicine Research Unit; NGOs: non-governmental organizations.

**Table 3.** Summary of published documents discussing MDA before the interviews

Author, year	Title	Content on MDA	Characteristics of published literature
Atkinson et al., 2011^32^	The architecture and effect of participation: a systematic review of community participation for communicable disease control and elimination; implications for malaria elimination	Systematic review focusing on the importance of community participation for communicable disease control and elimination (including MDA)	Accessible on PubMed
Poirot et al., 2013^21^	Review—Mass drug administration for malaria	Systematic review on MDA. This review concluded that MDA can quickly reduce malaria parasitaemia as well as improve several clinical outcomes. More studies are needed to assess its impact after 6 months. Furthermore, the barriers for community uptake as well as the potential contribution to the development of drug resistance need to be explored	Accessible on PubMed
Newby et al. 2015^19^	Review—Mass drug administration for malaria and its operational challenges	Systematic qualitative review of published, unpublished and grey literature documenting past MDA experiences. Information was also acquired through consulting with field experts, exploring their historical experience to provide an informed and contextual perspective on the role of mass drug administration in malaria elimination. Knowledge gaps remain and more research is necessary, particularly on the optimal size of the target population, primaquine safety and effective methods to improve population coverage. Despite these gaps, MDA has been shown to be successful in controlling and eliminating *Plasmodium falciparum* malaria as well as *Plasmodium vivax* malaria. MDA should therefore be considered as part of a comprehensive malaria elimination strategy in specific settings	Accessible on PubMed
von Seidlein et al. 2015^1^	Review—Fighting fire with fire: mass antimalarial drug administrations in an era of antimalarial resistance	Review on MDA. Recent emergence of artemisinin-resistant *P. falciparum* is of greatest concern. If ongoing efforts to contain artemisinin resistance are successful, it has yet to be shown. Currently, with no other promising plans to eliminate *falciparum* malaria from foci of artemisinin resistance, using a multipronged approach including MDA has been suggested. MDA is controversial, as it may increase drug pressure. It is difficult to conceptualize how TME may contribute to increase artemisinin resistance, provided a full treatment course is given	Accessible on PubMed
2015	WHO Recommendations on MDA (2015, 2017) (http://www.who.int/malaria/publications/atoz/role-of-mda-for-malaria/en/)	The recommendations are based on a recent review of evidence (Mass drug administration, mass screening and treatment and focal screening and treatment for malaria. WHO Evidence Review Group meeting report, WHO Headquarters, Geneva, 20–22 April 2015) and the advice of the Malaria Policy Advisory Committee	Accessible on the WHO homepage
2017	Operational Manual on MDA for *falciparum* malaria (2017) (http://www.who.int/malaria/mpac/mpac-mar2017-MDA-draft-manual-session7-presentation.pdf)	Manual on practical strategies for MDA for *falciparum* malaria	Accessible on the WHO homepage
Adhikari et al. 2016^16^	Review—Community engagement and population coverage in mass anti-malarial administrations: a systematic literature review	Review built on a previous review that identified 3049 articles describing MDAs published between 1913 and 2011. A total of 51 articles were retained for analysis, describing population coverage and/or community engagement in mass MDAs. Population coverages were quantitatively assessed and a thematic analysis explored community engagement activities. Further research is needed to better understand the factors that influence adherence and population coverage in MDAs and the role of community engagement in satisfactory participation	Accessible on PubMed

Despite the evidence regarding the importance of the sub-clinical parasite reservoir and the potential strategies to address this, the concept of asymptomatic malaria/sub-clinical malaria reservoir is still a relatively new concept for many policy makers. Moreover, policy makers are often guided by national malaria reports that focus on the incidence of clinical cases as a key indicator of success. For them, addressing asymptomatic cases therefore might be a low priority.

Other challenges, for example in Myanmar, included policy makers seemingly misunderstanding the TME pilot studies as a malaria control strategy.^35^ This delayed the pilot studies and the generation of evidence regarding the effectiveness of this approach.

The WHO’s initial hesitation to support MDA as a tool for malaria control was reflected by the decision to oppose MDA made by several countries in the GMS, even at an experimental stage. Following the WHO’s 2015 supportive recommendations to include MDAs as a malaria control and elimination tool,^33^ more countries began to consider MDA as a potential tool for malaria control programmes.

### Politics and the legacy of past MDAs

Both researchers and policymakers recalled their past experiences of MDAs and acknowledged the challenges in the field. The tragedy of an unpublished 2008–9 MDA in Cambodia, during which hundreds of patients were hospitalized, influenced the attitudes of policymakers and caused significant delays to the TME pilot study in that country.^22^ Recently conducted MDAs in the GMS that used DHA-PPQ as an antimalarial did not show any cardiotoxicities.^12–14^ A recent systematic literature review on the safety and efficacy of DHA-PPQ echoed these findings.^15^ Negative experiences of past MDAs for other diseases also influenced attitudes in target communities.^36^

Politics played a role among policymakers, for example in Myanmar and Vietnam, where opposing parties’ objections led to delays in approval of the pilot studies. Political divisions within target communities were also relevant; for example, close to the Thai–Myanmar border, where political affiliations prevented half of the villagers from participating in the MDA.^29^

### Choosing appropriate sites for MDA

Many potential sites for MDA in the GMS include populations that are mobile. This has been identified as a critical factor affecting the coverage of MDA and its impact of transmission.^19,21,36,37^ On Aneityum Island, where MDA for malaria elimination was successful, a rigorous system of detection and treatment of all persons coming to the island was attributable to its success.^38,39^ In settings where population movements can be monitored, for example in remote highlands and small islands, MDA is more likely to have a sustained impact on malaria transmission than in well-connected areas.^19,21^ These findings and the fact that many malaria endemic settings are often located in remote areas within the GMS, policymakers might have prompted doubts about the feasibility, uptake and potential benefits of MDA. Given that MDA requires an intensive approach to achieve high population coverage and adherence, the challenges for policymakers of implementing MDA in such settings might have outweighed the benefits of testing both a pilot TME study^12,13^ and its roll out.^14^

It was generally agreed that MDAs would only be effective in low transmission areas.^19,21,36^ However, working in these locations often involves many challenges: accessibility due to distant location, bad or non-existing roads, seasonal flooding, etc.^22,25^ In these remote populations, additional challenges include low levels of literacy, lack of primary health care, and language and communication issues.^16,22,23,25–29,32,35^

The importance of choosing the right season for MDA is a major factor. The best time of year to implement MDA is generally considered to be during or after the rainy season, when malaria transmission is high, as highlighted in a recent MDA from Cambodia.^22,24^ However, in settings where the agricultural workload is largely correlated with the rainy season,^22,25^ an MDA can interrupt the work of community members and may not receive a high priority. Accessibility to these villages because of poor road conditions and flooding is even worse during the rainy season.^25,28,29^ In Cambodia, several villagers suffered from seasonal flu but attributed the symptoms to the MDA drug they had taken. This subsequently had a strong negative impact on participation.^22^

### Strengths and limitations

Using a snowball approach to recruit participants facilitated access to difficult-to-reach policymakers involved in malaria prevention and control in the GMS and key decision makers at large international funders. Although such an approach has the potential to bias the make up of the study respondents (e.g. they might represent a particular subgroup of scientists with a particular perspective on MDA), disagreement among respondents, particularly regarding the key issue of whether MDA has the potential to promote resistance, suggested that this was not the case.

## Conclusions

Although most respondents identified implementation challenges related to MDA, national and international policymakers were particularly sceptical about applying this strategy for malaria elimination. The complex rationale of MDA for malaria elimination, mistaking pilot studies for implementation, past experiences with MDA, difficulties in selecting appropriate sites and the WHO’s lack of clear backing undermined support for this approach among policymakers. The WHO’s role was pivotal and its change in stance towards MDA for malaria elimination prompted a change in the opinions of national policymakers.

## Authors’ contributions

NK designed the study and collected data, supervised by CP and PYC. NK and BA analysed the data, supervised by CP, and wrote the first draft supervised by CP. All other authors read and approved the final manuscript.

## Acknowledgements

The authors would like to sincerely acknowledge Prof Arjen M. Dondorp for overall supervision of this study. We also thank Dr Lorenz von Seidlein for constructive feedback on the manuscript. We thank the study participants and communities, village malaria workers and local authorities. We also thank the TME community engagement team. The datasets generated and analysed during the current study are available via a Data Access Committee (see http://www.tropmedres.ac/data-sharing) complying with the data access policy (http://www.tropmedres.ac/_asset/file/data-sharing-policy-v1-0.pdf).

## Funding

The Mahidol Oxford Tropical Medicine Research Unit is funded by the Wellcome Trust of Great Britain (reference 101148/Z/13/Z). This study was funded by a bursary awarded by the Global Health Bioethics Network, which is funded by a Wellcome Trust Strategic Award (096527).

## Competing interests

None declared.

## Ethical approval

Approval was obtained from the National Ethics Committee for Health Research Cambodia (NECHR 0042 and 0051) and the Oxford Tropical Research Ethics Committee (OXTREC; 1017-13) and the study was registered on clinicaltrials.gov (NCT01872702). Written informed consent was obtained from all TME study participants or from the parent or guardian of minors. Verbal consent was obtained prior to interviews and was audio recorded.
